# Value-based genomics

**DOI:** 10.18632/oncotarget.24353

**Published:** 2018-01-30

**Authors:** Jun Gong, Kathy Pan, Marwan Fakih, Sumanta Pal, Ravi Salgia

**Affiliations:** ^1^ Department of Medical Oncology, City of Hope National Medical Center, Duarte, CA, USA; ^2^ Los Angeles Biomedical Research Institute at Harbor-UCLA Medical Center, Torrance, CA, USA; ^3^ Medical Oncology and Experimental Therapeutics, City of Hope Comprehensive Cancer Center, Duarte, CA, USA

**Keywords:** next-generation sequencing, precision oncology, pathways, cost-effectiveness, value-based care

## Abstract

Advancements in next-generation sequencing have greatly enhanced the development of biomarker-driven cancer therapies. The affordability and availability of next-generation sequencers have allowed for the commercialization of next-generation sequencing platforms that have found widespread use for clinical-decision making and research purposes. Despite the greater availability of tumor molecular profiling by next-generation sequencing at our doorsteps, the achievement of value-based care, or improving patient outcomes while reducing overall costs or risks, in the era of precision oncology remains a looming challenge. In this review, we highlight available data through a pre-established and conceptualized framework for evaluating value-based medicine to assess the cost (efficiency), clinical benefit (effectiveness), and toxicity (safety) of genomic profiling in cancer care. We also provide perspectives on future directions of next-generation sequencing from targeted panels to whole-exome or whole-genome sequencing and describe potential strategies needed to attain value-based genomics.

## INTRODUCTION

Although often interchanged with personalized medicine, the term precision medicine was first given prominence by the National Research Council with the intention to convey a broader concept that although treatments were rarely developed for individuals, subgroups of patients could be defined and targeted by genomics [1]. The concept of precision medicine in oncology further rose to popularity with the launching of the Precision Medicine Initiative aimed to accelerate development of therapeutic strategies that are biomarker-driven [2].

Over the past several decades, many major advances in cancer treatment have already been attributed, in part, to precision medicine or biomarker-driven treatment. Perhaps one of the earliest examples of biomarker-driven therapy was the development of tamoxifen in the adjuvant treatment of hormone-receptor positive breast cancer [3–5]. The approval of the break point cluster (BCR)-Abelson (ABL) tyrosine-kinase inhibitor (TKI) imatinib for chronic myelogenous leukemia (CML) in 2001 heralded the era of targeted therapy [6–8]. More recently, several clinical trials in oncology have launched to match targeted therapy to genomic profiling including the Signature, I-SPY, National Cancer Institute (NCI)-sponsored NCI-MATCH, NCI-MPACT, ALCHEMIST, Lung-MAP, Pediatric MATCH, and Exceptional Responders, Lung Cancer Mutation Consortium (LCMC)-sponsored, and American Society of Clinical Oncology (ASCO)-sponsored TAPUR trials [9–17]. In the United Kingdom, Innovate UK has recently launched an initiative to support €6 million in the development of precision medicine technologies that can lead to the improvement in targeted therapies [18].

Paramount to the advancement of precision oncology, the development of next-generation sequencing (NGS) techniques with high-throughput functionality has enabled the massive parallel sequencing of genomes at unprecedented rates [19]. With the ability to rapidly and efficiently perform genomic sequencing, many Clinical Laboratory Improvement Amendments (CLIA)-certified commercial and academic laboratories have developed validated NGS platforms with targeted panels that are increasingly being incorporated into clinical-decision making and cancer research [20]. However, total costs of cancer care are rapidly increasing, of which only 5–20% are accounted by cancer drugs, and are projected to increase in the U.S. from 125 billion dollars in 2010 to 158 billion dollars in 2020 [21]. Furthermore, costs of cancer care including copayments, out-of-pocket expenses, and insurance premiums often exceed patients’ expectations and impose significant financial stress and burden that can adversely affect quality of life (QOL) and outcomes [21, 22].

In the era of precision oncology, improving patient outcomes while reducing overall costs or risks with the incorporation of NGS is paramount to achieving value-based care [23, 24]. In this review, we provide a brief historical context on the evolution of NGS platforms to its current most widely-used commercial panels and compare specifications, mutation coverage, and costs of the more popular commercial platforms. In particular, we review our necessary and sufficient biomarkers that have come to clinical fruition. Through pre-established frameworks for evaluating value-based medicine, we highlight available data to assess the cost (efficiency), clinical benefit (effectiveness), and toxicity (safety) of genomic profiling in cancer care. We also provide perspectives on future directions of NGS from targeted panels to exomes/genomes and illustrate potential important strategies needed to attain value-based genomics.

### Historical perspective on next-generation sequencers

The completion of the human genome sequencing project in 2003 paved way for investigations focused on deciphering the human cancer genome [25]. The technology that sequenced the human genome was based on traditional Sanger sequencing and required about a decade of multicenter collaboration, automated analysis, and hundreds of millions of dollars [26, 27]. In the ensuing years, a paradigm shift occurred with the development of NGS techniques that accelerated the capability to analyze the cancer genome at speeds far superior than first-generation Sanger sequencing platforms [26, 28–30].

The commercialization and availability of these next-generation sequencers including those from Roche, Life Technologies SOLiD, Illumina, Pacific Biosciences, and Ion Torrent have since established the foundation for which most commercial NGS platforms operate from in tumor molecular profiling [27, 29, 31, 32]. The evolution of next-generation sequencers and comparisons of the technical specifications and costs across individual sequencers have been extensively reviewed [27, 29–32].

Currently, NGS technology can be broadly employed for 2 types of sequencing: targeted sequencing of a panel of recognized or putative cancer-associated genes and whole-exome or whole-genome sequencing (WES/WGS) for clinical-decision making, research purposes, and/or discovery of disease-causing genomic alterations [33]. The preference for targeted gene panels or WES or WGS remains a controversial topic of discussion in precision oncology. Large targeted panels were initially lauded for their improved efficiency, sensitivity, and ability to detect rare and potentially actionable genomic variants across tumor types compared to traditional smaller or multiple single-analyte gene panels [34]. Targeted panels have also been proposed as cost-effective and more readily interpretable than WES, while WES provides comprehensive profiling of all protein-encoding genes of the genome that can provide more information and long-term cost-effectiveness, over the life of the individual [35].

Nevertheless, gene panels are currently recommended by national guidelines such as the National Comprehensive Cancer Network (NCCN) in the diagnostic evaluation and management for several cancers and are recognized as a more pragmatic NGS-approach in universal healthcare systems [36, 37]. In the current state, targeted panels are more regularly used in cancer diagnostics while WES and WGS are not yet as incorporated into routine clinical practice in cancer care [38]. Cancer treatment pathways represent an increasingly important and popular mechanism to consolidate the massive amount of molecular data offered by NGS and patient care. Here, clinical pathways have been shown to facilitate the delivery of high-value cancer care by helping oncologists identify evidence-based treatments of greatest clinical benefit while reducing costs and likely represent a viable strategy for cost-effective precision oncology in the present and foreseeable future [23, 39, 40].

### Defining and measuring value in precision oncology

In its purest sense, value in oncology is defined as a measure of outcome per monetary expenditure where cancer therapies of high value lead to significant improvements in patient health outcomes at relatively low overall cost or risk [24, 41]. The Institute of Medicine (IOM) identified 6 elements of value in cancer care: effectiveness, safety, patient-centeredness, efficiency, timeliness of therapy, and equity [42]. Along similar lines, many oncology professional societies, government-sanctioned entities, and institutions across the globe have developed assessment tools and measures to evaluate the value of new therapies and health technologies in cancer care [41, 43–45]. In 2015, the ASCO Value in Cancer Task Force established their own framework and defined value in cancer care by clinical benefit (effectiveness), toxicity (safety), and cost (efficiency) with an update in 2016 [41, 45]. Although the ASCO task force recognized all 6 elements from the IOM’s definition of value-based medicine, their framework in defining value in cancer care emphasizes the 3 elements that are believed to be more readily measured and reported as outcomes in clinical trials and therefore ascertainable from high-quality medical evidence.

### A framework for discussion of value-based genomics

To the best of our knowledge, discussions on value-based genomics, for which we define as the value of genomic profiling in cancer care through the current availability of NGS platforms, are relatively novel and limited compared to the growing discussions on value-based medicine focused on cancer drugs themselves. A similar conceptualized approach to assessing value in cancer genomics based on the ASCO framework is worthwhile to pursue and will be presented.

### Cost and comparison of commercial targeted gene panels

Next-generation sequencers became substantially more cost-effective with the authorization of the first sequencer by the U.S. Food and Drug Administration (FDA) in 2013 [46]. As a reference, to completely sequence the human genome and generate the first reference human genome sequence (about 3 billion bases of DNA), an extensive and global collaborative effort to produce a finished sequence of high fidelity was required with costs approximating hundreds of millions of dollars [47]. The Human Genome Project, in its entirety, took more than a decade to complete and produced a total cost of approximately 2.7 billion dollars [47]. With the reference human genome in hand and development of NGS technologies, sequencing an individual’s “personal” genome was a more readily attainable endeavor though with costs ranging from about 14–25 million dollars by 2006 [47]. At the time of the FDA approval of the first next-generation sequencer in 2013, sequencing a human genome could be performed in about less than 24 hours and under $5,000 [46]. Based on data collected by National Human Genome Research Institute (NHGRI)-funded genome-sequencing groups, the current cost to generate a whole-genome sequence is around $1,500 (less for whole-exome) with variation above and below this number based on commercial pricing [47].

The affordability and availability of these next-generation sequencers have allowed for the commercialization of a growing number of CLIA-certified laboratories offering tumor genomic profiling by NGS. Excluding targeted gene panels derived by individual academic institutions, there are several commercial targeted panels that have found widespread use for clinical-decision making and research purposes. To enhance cost-effectiveness and time-efficiency, commercial NGS platforms have developed targeted enrichment methods to capture and sequence only genomic regions of interest including amplification- and probe-based enrichment methods or hybridization [48, 49]. Multiple targeted NGS platforms are commercially available (Table 1) with several having undergone validation or evaluation in the clinical setting [50–57]. All platforms aimed at detecting somatic mutations utilize formalin-fixed, paraffin-embedded (FFPE) tumor tissue [58–65]. Companies such as Caris Molecular Intelligence perform analyses on fresh tissue or malignant fluid as well [59]. In general, the minimum required size of the sample is 5 mm^2^ (25 mm^2^ preferred) and the required ratio of tumor nuclei to benign nuclei is 20% [58]. Paradigm Cancer Diagnostic requires a minimum sample of 10 × 2 × 1 mm (20 mm^3^) with minimum 5% tumor content [62]. In addition to FFPE tumor samples, a few platforms analyze DNA from matched blood and/or saliva. Examples include OncoDNA and OncoSTRAT&GO [66] which performs genomic profiling on the tumor sample as well as on circulating tumor DNA, and Tempus xT and xO [61], which utilize blood or saliva samples to examine normal reference DNA. Ambry Genetics TumorNext uses paired blood-tumor samples to identify germline and somatic mutations in homologous recombination repair genes that predict response to PARP inhibitors [67].

**Table 1:** Selected commercially available targeted next-generation sequencing platforms and specifications

	Sample reqs	Sequencer	Genes covered and mutation types (if specified*)	Additional analyses	Cost^+^	Time	Year released	Ref.^^^
FoundationOne (Foundation Medicine	-FFPE -Prefer no decalcification, but may use EDTA	Illumina	315 genes (+28 introns) fusions, copy number variations	MSI TMB	5800 USD	14 days	2011	[50, 57]
Caris Molecular Intelligence (Caris Life Sciences	-FFPE -Fresh specimen in 10% neutral buffered formalin -Malignant fluid up to 120 cc -No decalcified specimens	Ilumina	>600 genes fusions, copy number variations	IHC MSI TMB ISH	6500 USD	10–14 days	2014	[51, 56]
OncoDEEP (OncoDNA)	-FFPE -Prefer no decalcification, but may use EDTA	Ion Torrent	75 genes fusions, methylation, splice variants	IHC MSI TMB	∼3500 USD (2990 Euros)	7 days	2014	[52]
OncoSTRAT& GO (OncoDNA)	-FFPE **and** 2 10 mL blood samples (for ct DNA)	Ion Torrent	> 500 genes (solid portion) + 40 genes (liquid portion) fusions, methylation, splice variants	IHC MSI TMB	∼5800 USD (4990 Euros)	10 days	2016	
Tempus xT/xO (Tempus Labs	-FFPE **and** matched blood (solid tumors) or saliva (lymphoma) sample for normal DNA -Prefer no decalcification, but may use EDTA	Illumina	595 genes (xT); 1711 genes (xO) fusions, copy number variations, splice variants	MSI TMB	4800 USD (Tempus xO)	14–21 days	2017	[54, 55]
Paradigm Cancer Diagnostic –PCDx (Paradigm	-FFPE	Ion Torrent	186 genes fusions, copy number variations, splice variants	IHC MSI	4800 USD	5 days	2014	[53, 57]
Oncomine Dx Target Test (Thermo Fisher Scientific	-FFPE	Ion Torrent	23 genes (NSCLC only)	-	-	4 days	2017	
OncoVantage Solid Tumor Mutation Analysis (Quest Diagnostics)	-FFPE	Ion Torrent	34 genes point mutations and indels only; **no** large rearrangements or copy number changes	-	1800–3000 USD [73]	14 days	2014	
**Hereditary Cancer Panels**								
OncoGeneDx Comprehensive Cancer Panel (GeneDx)	-2–5 mL blood -oral rinse or buccal swab as alternative	Illumina	32 genes (tumor-specific panels also available)	-	-	21 days	2013	
TumorNext-HRD (Ambry Genetics	-3–5 cc blood **and** FFPE	Illumina	11 genes (mutations in homologous recombination repair genes, in ovarian cancer only)	-	-	21–28 days	2017	
CancerNext (Ambry Genetics	-6–10 cc blood -1 cc saliva	Illumina	34 genes (tumor-specific panels also available; expanded 67 gene panel also available)	-	∼5830 USD	14–21 days	2012	

*Point mutations, insertions/deletions (indels) are covered unless otherwise stated.

^+^Prices are obtained from company websites or personal communication unless otherwise specified.

^^^References provided for clinical validation studies, when available, for specific platform.

FFPE, formalin-fixed, paraffin-embedded; EDTA, ethylenediaminetetraacetic acid; IHC, immunohistochemistry; MSI, microsatellite instability; TMB, tumor mutation burden; ISH, in-site hybridization; USD, United States dollars; NSCLC, non-small cell lung cancer.

The vast majority of panels utilize either Illumina or Ion Torrent sequencing (Table 1). With the exception of Quest OncoVantage [64, 65], most contemporary panels identify not only point mutations and insertions/deletions but also fusions/translocations and copy number variations. Many report microsatellite instability and tumor mutation burden to assist in identifying patients who may benefit from immunotherapy [58–61, 66]. In addition, many platforms incorporate results of immunohistochemistry (IHC) testing [59, 60, 62, 66]. The current movement is to not only look at the genomics but also proteomics. In terms of proteomics, IHC for PD-L1 has become standard in non-small cell lung cancer.

Turnaround time ranges from 4 days for Oncomine Dx, a 23-gene panel of lung-cancer related genes [63], to 21 to 28 days for Ambry Genetics TumorNext, which examines hereditary and somatic mutations in ovarian cancer patients [67]. Costs for testing are in the $3000 to 6000 range where pricing information is available. Head-to-head comparisons of the various platforms are sparse. Weiss *et al.* compared Foundation One and Paradigm Cancer Diagnostic (PCDx) using tumor samples from 21 patients and reported a faster turnaround time for PCDx and significant discrepancies in detection of actionable targets between the two platforms [57].

ASCO has deemed both the Foundation Medicine and Caris Life Sciences platforms “optimized” for reporting for the Targeted Agent and Profiling Utilization Registry (TAPUR) study [68]. This non-randomized clinical trial aims to examine the use of targeted therapy for patients with advanced, progressive cancer who are found to have actionable variants on genomic testing. Currently, the Oncomine Dx Target Test and the FoundationFocus CDxBRCA Assay are the only commercial NGS platforms that are approved by the FDA for use as companion diagnostic devices [69].

Although early in its infancy, there is a growing body of research focused on total costs and cost-effectiveness analyses of cancer-related genome sequencing in the era of NGS (Table 2). An early cost analysis of WGS through the Illumina HiSeq 2000 platform considered total expenses through a 4-step process: sample collection and experimental design, sample sequencing, data reduction and management, and downstream analyses [70]. In 2011, the estimated cost to perform WGS considering only the first 3 steps was $6,500 per assay over an approximate timeframe of 15 days. However, the authors forewarned that the rapid decreases in costs seen in data generation offered by advancing NGS technologies has not been matched by decreases in costs of the computational infrastructure needed to mine and manage the huge volume of data. Here, costs of downstream analyses were estimated to be onwards of an additional $100,000 per assay and requiring months to process generated data. A systematic review of 5 fully-published studies at the time provided a cost analysis on cost per megabase (Mb) for using various NGS platforms but concluded that the results were very heterogeneous and questionable regarding their reliability and validity given the unclear methodology and numerous costs that may have been unaccounted for across studies [71]. In short, health economic evidence for genome sequencing was limited at the time of the review, and a comprehensive calculation of genomic sequencing considering multiple aspects of cost is needed. Similarly, a systematic review of included studies from 2009–2014 highlighted that NGS platforms were less costly than Sanger sequencing but concluded that the lack of randomized control trials (RCTs) investigating the cost-effectiveness of NGS limited the ability to conduct an informed cost-effectiveness analysis [72].

**Table 2:** Summary of studies investigating the costs and cost-effectiveness of next-generation sequencing in cancer

Objective	Platform	Findings	Ref
Microcosting analysis	WGS, Illumina HiSeq 2000	Estimated $6,500 per case over a period 15 days for sample collection and experimental design, sample sequencing, and data reduction and management; for downstream analyses, an estimated additional >$100,000 per case requiring months	[70]
Systematic review of cost analyses	WGS/WES, various platforms	Variable cost per Mb ranging from <$0.07-$84.39/Mb and cost per sequencer ranging from $155,000-$1,350,000 per instrument depending on study	[71]
Systematic review of cost-effectiveness	WGS/WES/TGS, various platforms	Compared to Sanger sequencing (approximately $500/Mb), cost was less for NGS platforms (as low as $0.10/Mb) but unable to perform informed analysis of the cost-effectiveness of NGS given insufficient high-quality evidence	[72]
Cost-effectiveness analysis	34-gene NGS panel vs. single-site *BRAF*^V600^ test	Cost: $128,965 vs. $120,022 per patient (over 2-year time horizon) QALYs: 0.721 vs. 0.704 per patient (incremental 0.0174 QALYs with NGS over single-site testing over a 2-year time horizon)	[73]
Cost-effectiveness analysis	NGS panel vs. sequential evaluation for Lynch syndrome (SOC)	Compared to SOC, NGS panel resulted in an average increase of 0.151 year of life, 0.128 QALY, and $4,650 per patient (ICER of $36,500 per QALY with 99% probability of being cost-effective at $100,000 per QALY threshold)	[74]
Cost-effectiveness analysis of returning IFs	Receiving IFs vs. not receiving IFs from 56-gene NGS panel	For CRC patients, receiving IFs would increase costs by $2.9 million and increase QALYs by 25.4 years (ICER of $115,020) with <$100,000/QALY gained 28%	[75]
Microcosting and cost-impact analysis	5–50-gene and >50-gene NGS panel, WES	Estimated total costs: $577.99-$907.82 (5–50 genes), $1948 (>50 genes), $1499.32-$3388.18 (WES per case Cost-impact analysis for NGS implementation: Costs of targeted therapy increased from $1.1 million to $2.3 million, nontargeted therapy decreased from $8.8 million to $2.2 million, clinical trials increased by $2.7 million, and hospice care increased by $60,000; total cost of treatment decreased from $10.2 million to $7.5 million over a 6-month time horizon from diagnosis of advanced NSCLC and cost of genetic testing increases by $0.13 million (assuming $700 for 5–50-gene panel)	[76]
Microcosting and budget-impact analysis	48- and 178-gene NGS panel, Illumina Miseq or Hiseq	Estimated total costs per sample: €606–956 (48 genes), €1,137–2,668 (178 genes) Budget-impact analysis for incorporation of NGS in Netherlands: Annual increase of €1,321,243 (2012–2015) and annual decrease of €120,473 (2020, projected due to more efficient use of WGS) for stage IV NSCLC, annual increase of €108,526 (2012–2015) and €351,799 (2020, projected due to more widespread use of NGS in hospitals) for stage IV melanoma	[77]
Retrospective cost analysis	96-gene NGS panel vs. SOC genomic testing only	Total costs (includes patient treatment, toxicity, sequencing, and targeted drug therapy): $91,790 vs. $40,782 per patient (*p* = 0.002) Drug costs: $59,259 vs. $20,189 per patient (*p* < 0.001) Patient charges per week: $4,665 vs. $5,000 per week (*p* = 0.126) given that PFS 22.9 weeks (NGS group) vs. 12.0 weeks (SOC group, *p* = 0.002) with a HR of 0.47 (95% CI 0.29–0.75)	[78]
Time-and-motion microcosting analysis	Digital GEP vs. FISH vs. 32-gene targeted NGS	Mean per-case cost (assumes 180 cases annually, in Canadian dollars): $898.35 vs. $596.60 vs. $1,029.16 (NGS includes bioinformatics analysis) Labor-intensiveness: 258.2 minutes/case (FISH), 124.1 minutes/case (NGS, and 14.9 minutes/case (GEP)	[79]
Cost-effectiveness analysis	48-gene NGS panel and targeted therapy (off-label or clinical trial), no NGS and chemotherapy, no NGS and BSC	Life-years: Additional 0.009 LYs gained with NGS than chemotherapy or BSC (1.458 LYs) resulting in ICER of AUD 485,199/QALY Chemotherapy produced gain of 0.001 QALYs when compared to BSC (ICER of AUD 361,580/QALY) NGS produced gain of 0.008 QALYs when compared to chemotherapy (ICER of AUD 489,338/QALY)	[80]
Prospective microcosting analysis	50-gene NGS panel guiding targeted therapy (biomarker-based) vs. targeted therapy without NGS (biomarker-agnostic) vs. BSC	Estimated total cost-per-patient for months (includes drug costs, outpatient visits, costs from management of AEs and/or procedure complications, and sequencing): €9,654–16,798 vs. €29,870–37,707 vs. €4,147–13,889	[81]
Microcosting analysis	WGS, Illumina HiSeq 2500 and HiSeq Xten	Estimated overall costs per case (includes direct medical costs and site-specific costs for sequencing devices): €3858.06 (HiSeq 2500) and €1411.20 (HiSeq Xten)	[82]
Microcosting and forecast analysis	WGS, Illumina HiSeq 2500 (including RNA sequencing)	Estimated total costs per patient (2012–2015): $34,886 (95% CI $34,051–$35,721). 10-year forecast: WGS and RNA sequencing costs will reach $5000/patient by December 2019, $3000 by November 2020, and $1000 by September 2021.	[83]
Microcosting analysis	90-gene NGS panel (NextSeq500), WES (HiSeq4000) vs. WGS (HiSeqX5), all Illumina	Estimated total costs per sample (includes capital costs, maintenance costs, and operational costs over 5 year life cycle): €332.90 vs. €791.75 vs. €1669.02	[84]

WGS, whole-genome sequencing; WES, whole-exome sequencing; Mb, megabase; TGS, targeted gene sequencing; NGS, next-generation sequencing; QALYs, quality-adjusted life-years; SOC, standard of care; ICER, incremental cost-effectiveness ratio; IFs, incidental findings; CRC, colorectal cancer; NSCLC, non-small cell lung cancer; PFS, progression-free survival; HR, hazard ratio; CI, confidence interval; GEP, gene expression profiling; FISH, fluorescence *in situ* hybridization; BSC, best supportive care; LYs, life-years; AUD, Australian dollar; AEs, adverse events.

A later study incorporated a prediction model over a 2-year time horizon and performed a cost-effectiveness analysis of a 34-gene NGS panel vs. single-site BRAF^*V600*^ mutation testing in metastatic melanoma patients (Table 2). The NGS panel was shown to be less costly (saving $8,943 per patient) and was more effective than the single-site test [73]. Furthermore, sensitivity analyses showed that the NGS panel had a 90.9 % chance of having reduced costs and increased quality-adjusted life-years (QALYs) than single-site testing, and these findings would equate to an annual savings of $79.6 million and a gain of 155 QALYs if applied to 8,900 metastatic melanoma patients. One study applied a decision model for patients referred to the medical genetics clinic for hereditary colorectal cancer and polyposis (CRCP) syndromes to compare the cost-effectiveness of a NGS panel including Lynch syndrome genes and other genes associated with CRCP syndromes of high penetrance to standard of care (SOC) or sequential evaluation of Lynch syndrome genes recommended by current guidelines [74]. The NGS panel strategy was highly cost-effective below the contemporary $100,000 per QALY threshold, when compared to SOC, and this was among the first studies to model NGS panels as a cost-effective strategy in the evaluation of a relatively common inherited cancer syndrome (Table 2).

A decision model of returning incidental findings (IFs) detected by a 56-gene NGS panel for patients with cardiomyopathy, colorectal cancer (CRC) or polyposis, and in healthy individuals referred for testing given family history and risk factors showed that returning IFs may be cost-effective for certain populations with incremental cost-effectiveness ratios (ICERs) of $44,800, $115,020, and $58,600, respectively [75]. Notably, primary screening of healthy individuals (assuming NGS costs of $500 per test) was not cost-effective with an ICER of $133,400 (<$100,000/QALY gained in only 10% of simulations). A recent study provided a microcosting analysis for targeted NGS panels and WES as well as a cost-impact analysis for patients diagnosed with advanced non-small cell lung cancer (NSCLC) employing NGS for optimization of first-line treatment (Table 2). A before and after comparison (time horizon of 6 months from diagnosis) showed that adopting NGS into management increased use of targeted therapy (6% to 13%), decreased use of nontargeted therapy (83% to 20%), decreased adverse events (AEs) from 207 to 138 (for a plan size of 1 million members), increased enrollment in clinical trials (4% to 54%), and increased referral to hospice care (7% to 13%) [76]. A Netherlands study provided a budget-impact analysis for implementation of NGS (48- and 178-gene panels) in stage IV NSCLC and melanoma patients within the Dutch health care system and demonstrated that the more samples that are run, the less costly NGS will become [77]. The budget analyses were comparable to a case study of 172 stage IV NSCLC and melanoma patients within the Netherlands Cancer Institute, and in a before and after NGS implementation comparison, there were no significant difference in treatment characteristics and clinical trial enrollment though an increased number of observed mutations in both cohorts was not surprisingly observed.

A retrospective study of 72 patients with treatment-refractory advanced solid tumors demonstrated increased total patient care costs (mainly contributed by costs of drug therapy) using a management strategy guided by a 96-gene NGS panel compared to standard of care genomic testing only approach (Table 2). However, the significantly improved progression-free survival (PFS) seen in the precision medicine group over the control group (22.9 weeks vs. 12.0 weeks, *p* = 0.002) translated to patient charges per week that were similar between both groups, suggesting that precision oncology potentially improves survival without increasing health care costs [78]. A time-and-motion microcosting analysis of 3 genomic assays (digital gene expression profiling (GEP), fluorescence *in situ* hybridization (FISH), and 32-gene targeted NGS panel) in the management of patients with non-Hodgkin lymphoma illustrated that FISH was found to be most labor-intensive followed by NGS and digital GEP, while targeted NGS with bioinformatics analysis had the highest mean per-case cost (in Canadian dollars) followed by digital GEP and FISH [79]. In an Australian cost-effectiveness analysis, the implementation of a 48-gene NGS panel leading to targeted therapy (off-trial or clinical trial setting) in the fourth-line treatment of metastatic NSCLC was found to have unfavorable ICERs (Table 2). The authors commented that reduction in off-label costs, lower mortality rates for true positive patients and during testing, lower health utility costs for progressive disease, and reductions in inpatient visits due to targeted therapy were all potential factors contributing to more favorable cost-effectiveness of a targeted NGS approach [80].

From 2014–2015, a Norwegian study prospectively enrolled 24 patients with refractory advanced solid tumors to 3 treatment strategies: best supportive care (BSC), targeted therapy without knowledge of a tumor biomarker (biomarker-agnostic), or targeted therapy guided by a 50-gene NGS panel (biomarker-based) and analyzed total cost-per-patient for each approach over 3 months [81]. Here, the biomarker-agnostic approach was approximately 2.5-fold more costly than the biomarker-based approach with the main drivers being drug costs and AE management. The biomarker-based approach was approximately 2.5-fold more costly than BSC and was mainly driven by costs of diagnostic procedures. A German study provided an overall cost analysis for WGS via Illumina platforms and showed that the latest HiSeq Xten was approximately 63% cheaper than the HiSeq 2500 platform (Table 2). Notably, the difficulty in the cost analysis for WGS was highlighted given the multitude of cost-influencing factors that were included and excluded in this study [82]. Furthermore, overhead costs should be considered to truly obtain a comprehensive cost analysis of WGS in the healthcare system. A separate British microcosting analysis of WGS over the 2012–2015 time period revealed higher median costs per patient likely due to apply both WGS and RNA sequencing over multiple samples to achieve a higher coverage rate of >80-fold than conventional 30-fold coverage [83]. Of note, 10-year forecasting scenarios did not produce expected WGS costs to reach $1000 per patient in the next 10 years. A Dutch comparison of 3 leading Illumina NGS platforms: NextSeq500 90-gene targeted panel, HiSeq4000 WES, and HiSeqX5 WGS similarly showed that per-sample costs for WGS did not reach the $1000 threshold [84]. Although the targeted panel and WES were considerably cheaper alternatives than WGS, the decision to apply a specific platform to clinical practice should consider potential tradeoffs between costs and expected clinical utility of the selected approach.

### Clinical benefit or effectiveness

The topic of whether precision oncology improves clinical outcomes has been hotly debated [85, 86]. Often cited in these discussions, is the prospective phase II SHIVA trial that did not show superior outcomes in those with treatment-refractory advanced solid tumors matched to molecular targeted therapy based on molecular profiling, when compared to control, though this study has often been criticized for serious issues in methodologic design [87–89]. Conversely, preliminary results from the prospective phase I MOSCATO 01 and MD Anderson trials demonstrated feasibility and promising antitumor activity of targeted therapy matched according to NGS of tumors in previously-treated patients with advanced solid tumors [90–92]. To highlight the potential clinical benefit of NGS-directed or biomarker-driven therapy in cancer, we limited our review to data from the largest series and meta-analyses (Table 3) and defer discussion of smaller published studies elsewhere [13, 56, 93–97]. Studies in lung cancer have often served as the prototype for assessing the clinical benefit of molecular profiling in cancer management, and a large retrospective analysis of 143 single-agent phase II trials from 2000–2009 in >7,000 advanced NSLCL patients demonstrated superior median overall response rate (ORR), PFS, and overall survival (OS) in trials enriched for the presence of molecular targets compared to studies with unselected patients [98]. On multivariate analysis, enrichment for putative molecular targets was an independent predictor for greater ORR, PFS, and OS.

**Table 3:** Summary of large series and meta-analyses evaluating the clinical benefit or effectiveness of molecular profiling in cancer

Design, *n*	*n*	Findings	Ref
Retrospective, 143 phase II trials	7, 701 advanced NSCLC patients	12 studies enriched for the presence of molecular targets had improved median ORR 48.8% (IQR 71, *p* = 0.005), PFS 6 months (IQR 6.8, *p* = 0,005), and OS 11.3 months (IQR 11.2, *p* = 0.05) compared to 9.7% (IQR 13.6), 2.8 months (IQR 1.9), and 7.5 months (IQR 3.2), respectively, in studies with unselected patients; enrichment for putative molecular targets was independent predictors of ORR, PFS, and OS on multivariate analysis (all *p* ≤ 0.005)	[98]
Meta-analysis, 112 FDA registration trials	38, 104 patients with various solid tumors and hematologic malignancies	In randomized trials (*n* = 57): Improved RRR 3.82 (95% CI 2.51–5.82, adjusted *p* = 0.03), longer PFS (HR 0.41, 95% CI 0.33–0.51, *p* < 0.001), and longer OS (HR 0.71, 95% CI 0.61–0.83, *p* = 0.07) with personalized therapy compared to RRR 2.08 (95% CI 1.76–2.47), PFS (HR 0.59, 95% CI 0.53–0.65), and OS (HR 0.81, 95% CI 0.77–0.85) with nonpersonalized therapy arms In experimental arms in all 112 trials: Personalized therapy had higher response rate (48%, 95% CI 42%–55% vs. 23%, 95% CI 20%–27%, *p* < 0.001), longer median PFS (8.3 months, IQR 5 vs. 5.5 months, IQR 5, adjusted *p* = 0.002), and longer median OS (19.3 months, IQR 17 vs. 13.5 months, IQR 8, adjusted *p* = 0.04)	[99]
Meta-analysis, 570 phase II trials	32, 149 patients with various solid tumors and hematologic malignancies	On multivariable analysis, personalized treatment approach (vs. nonpersonalized approach), had higher median RR (31% *vs.* 10.5%, *p* < 0.001), longer median PFS (5.9 *vs.* 2.7 months, *p* < 0 .001), and longer median OS (13.7 *vs.* 8.9 months, *p* < 0 .001) Nonpersonalized arms had poorer outcomes compared with personalized or cytotoxic arms with median RR of 4%, 30%, and 11.9%, respectively, median PFS of 2.6, 6.9, and 3.3 months, respectively (all *p* < 0.001), and median OS of 8.7, 15.9, and 9.4 months, respectively (all *p* < 0.05)	[100]
Meta-analysis, 346 phase I trials	13, 203 patients with various solid tumors and hematologic malignancies	Compared to a nonpersonalized approach, a personalized approach had higher median RR (30.6%, 95% CI 25.0%–36.9% vs. 4.9%, 95% CI 4.2%–5.7%, *p* < 0.001) and longer median PFS (5.7 months, 95% CI 2.6–13.8 vs. 2.95 months, 95% CI 2.3–3.7, *p* < 0.001) Nonpersonalized targeted arms had comparable outcomes to cytotoxic arms: Median RR 5.1% (95% CI 4.3%–6.0%) vs. 4.7% (95% CI 3.6%–6.2%, *p* = 0.63) and median PFS 3.3 months (95% CI 2.6–4.0) vs. 2.5 months (95% CI 2.0–3.7, *p* = 0.22), respectively	[101]

NSCLC, non-small cell lung cancer; ORR, overall response rate; IQR, interquartile range; PFS, progression-free survival; OS, overall survival; FDA, Food and Drug Administration; RRR, relative response rate; HR, hazard ratio; CI, confidence interval; RR, response rate.

A meta-analysis of 112 registration trials (57 randomized and 55 nonrandomized) from 1998–2013 leading to FDA drug approvals in cancer therapy compared efficacy outcomes between therapies employing a personalized treatment approach (matched targeted therapy) vs. those that did not [99]. In randomized registration trials and experimental arms of all registration trials, personalized therapy was associated with higher response rates, longer PFS, and longer OS compared to nonpersonalized therapy arms (Table 3). Furthermore, a personalized treatment strategy was an independent predictor of improved response rate, PFS, and OS on multilinear regression analysis.

A subsequent meta-analysis of 570 single-agent phase II trials from 2010–2012 similarly investigated efficacy outcomes in 32,149 patients with various cancers treated with a personalized and nonpersonalized treatment strategy [100]. Compared to a nonpersonalized approach, a personalized approach consistently and independently was associated with improved response rates, PFS, and OS (Table 3). Furthermore, nonpersonalized treatment arms had significantly poorer outcomes compared to either personalized or cytotoxic arms. Personalized arms employing a genomic biomarker had improved response rates, PFS, and OS (all *p* ≤ 0.05) compared to personalized arms using a protein marker.

A recent meta-analysis of 346 phase I trials from 2011–2013 evaluated the efficacy of biomarker-guided treatment selection (personalized) vs. treatment selection that was not biomarker-based (nonpersonalized) in 13,203 patients with solid tumors and hematologic malignancies [101]. Again, patients selected for treatment via a personalized approach had significantly improved median RR and PFS than those under a nonpersonalized approach (Table 3). Biomarker-based targeted therapy arms (*n* = 57 trials) correlated with significantly improved RR compared with targeted therapy arms (*n* = 177 arms) that were not biomarker-driven (31.1%, 95% CI 25.4%–37.4% vs. 5.1%, 95% CI 4.3%–6.0%, *p* < 0.001). Nonpersonalized targeted therapy arms had outcomes comparable to cytotoxic arms, and personalized arms using a genomic biomarker had improved median RR than those employing a protein biomarker (42.0%, 95% CI 33.7%–50.9% vs. 22.4%, 95% CI 15.6%–30.9%, *p* = 0.001).

Not surprisingly, genome sequencing is undergoing widespread implementation into routine cancer patient care (Supplementary Table 1). Molecular profiling is now incorporated into standard practice guidelines recommended by the NCCN while offering research value through facilitating the investigation of potential biomarkers of interest in breast [102–110], colorectal [111–115], gastroesophageal [116–122], hepatobiliary [123–127], pancreatic [128–130], gynecologic [131–143], prostate [144–152], other genitourinary (kidney, germ cell, and bladder) [153–166], lung [167–179], head and neck [180–187], melanoma [188–194], soft tissue sarcomas [195–204], and central nervous system cancers [205–212].

### Toxicity (including financial) and safety

In an early study assessing outcomes in 66 treatment-refractory metastatic cancer patients selected for therapy based on molecular profiling, safety analyses demonstrated no treatment-related deaths and 1 treatment discontinuation (1.5%) due to grade 2 fatigue using the molecular profiling treatment approach [56]. In a cost-impact analysis of advanced NSCLC patients employing NGS for optimization of first-line treatment, adaptation of NGS into management was shown to decrease the frequency of adverse events compared to the period prior to NGS incorporation [76].

In a meta-analysis of 112 registration trials leading to FDA-approved cancer therapies, the treatment-related mortality rate was 1.6% (95% CI 1%–2.4%) for trials employing a personalized strategy (matched targeted therapy) and similar to the 1.4% (95% CI 1%–2%, *p* = 0.74) for nonpersonalized trials [99]. That a personalized treatment approach was not more toxic than nonpersonalized treatment strategies was also shown in a meta-analysis of 346 phase I cancer trials where the median treatment-related mortality rate was 1.89% (95% CI 1.36%–2.61%) for arms using a personalized strategy and 2.27% (95% CI 1.97%–2.62%, *p* = 0.31) for arms without a personalized strategy [101]. A large meta-analysis of 570 single-agent phase II trials even demonstrated a lower median treatment-related mortality rate of 1.52% (95% CI 1.23%–1.87%) in personalized treatment arms compared to 2.26% (95% CI 2.04%–2.49%, *p* < 0.001) in nonpersonalized arms [100]. Further analysis confirmed that cytotoxic agents had higher median treatment-related death rates (2.42%, 95% CI 2.08%–2.83%) than targeted therapy arms (median 1.94%, 95% CI 1.74%–2.17%, *p* = 0.023). In addition to the potentially increased morbidity and mortality from treatment-related AEs in nonpersonalized strategies, AE management has been shown to be a main driver of cost and reason for greater expenses with therapies not based on biomarker strategies compared to biomarker-based approaches [81].

A recent meta-analysis of 41 randomized clinical trials evaluating 28 targeted agents for solid tumors approved by the FDA since 2000 evaluated the rate of treatment-discontinuation due to toxicity and grade 3–4 AEs and showed that targeted therapies with companion diagnostics were associated with improved safety and tolerability [213]. Specifically, agents with companion diagnostics compared to those without companion diagnostics had lower odds of treatment discontinuation (odds ratio (OR) 1.12 vs. 1.65, *p* < 0.001) and grade 3–4 AEs (OR 1.09 vs. 2.10, *p* < 0.001) with differences in safety being greatest for gastrointestinal, neurologic, and cutaneous toxicity. Indeed, the FDA has recently implemented a policy requiring the co-approval of a diagnostic with a therapeutic agent when the companion diagnostic is essential to the safe and effective use of the therapeutic product [214]. To achieve this, the FDA has executed numerous accommodations to facilitate this process without slowing the approval of the co-developed products.

Beyond the toxicities associated with cancer therapies, the financial toxicity associated with cancer care is becoming increasingly relevant in the face of rising cancer care costs and given that out-of-pocket expenses, copayments, and insurance premiums can often cause significant financial stress and burden to cancer patients that can adversely affect QOL and outcomes [21, 22]. Accordingly, evidence-based financial toxicity grading systems analogous to the NCI-Common Terminology Criteria for Adverse Events grading system have been developed and are undergoing validation [215].

A timely study has been conducted in recognition that tailoring cancer therapies to individual patients based on NGS is an emerging field that lacks formal coverage by the majority of U.S. payers [216]. Here, interviews of private payers covering more than 2/3 of the U.S. insured population provided perspectives and challenges that remain to NGS reimbursement. Of 7 senior executives from the 10 largest U.S. health plans and regional plans covering >125 million enrollees interviewed, 80% agreed that NGS has substantive potential to benefit and transform the state of cancer care. However, 80% of the panel agreed that NGS does not fit the definition of “medically necessary” and is considered “experimental or investigational.” One additional concern was that coverage in this instance may appear as an endorsement for novel targets and related off-label use. Notably, 40% considered a pan-cancer NGS application beneficial given that it is already common in oncology and provides rationale for off-label drug use, and although formal coverage for pan-cancer therapies may not be provided by payers, payment could be continued on exception bases. Furthermore, 70% of the panel recognized that NGS represents a misalignment to the “single test/single result” contemporary coverage approach, while 60% believed that the accompanying bioinformatics should be considered its own diagnostic for which there has been no precedent to pay for separately. For reasons including lack of large correlative studies and lack of experience with new study methodologies, 70% of the panel believe that the current evidence methods proposed for NGS do not fit payers’ evidentiary standards. Lastly, for reasons including potential for departure from standard care protocols, lack of transparency on NGS application, and lack of competent infrastructure, 50% of the panel raised concerns regarding the adoption and implementation of NGS in cancer care.

## DISCUSSION

In this review, we highlighted available data through the ASCO Value in Cancer Task Force framework to assess the value of genomic profiling in cancer care. Advancements in NGS technologies and our greater understanding of tumor molecular biology have, in part, led to achievements in cost (efficiency), clinical benefit (effectiveness), and toxicity (safety) in precision oncology. However, significant challenges remain and need to be considered in order to attain value-based genomics.

Genomic profiling has become undeniably more cost-effective since the time of the human genome sequencing project when the cost of sequencing the human genome ranged in the hundreds of millions of dollars and took over a decade to complete to current NGS platforms that can sequence an individual’s genome on the order of days with costs that range in the thousands of dollars [46, 47]. Several studies have shown the cost-effectiveness of NGS panels over single-analyte tests, over SOC sequential genetic testing, and in one instance, a NGS-guided treatment strategy showed improvement in survival without increasing healthcare costs compared to a SOC genomic testing only approach [73, 74, 78]. In another study, implementation of targeted therapy based on NGS in refractory metastatic NSCLC produced an unfavorable ICER suggesting that this approach was not cost-effective [80]. Several studies have shown that NGS panels and related management strategies are less costly than Sanger sequencing, targeted therapy approaches without NGS, or treatment of advanced NSCLC without incorporation of NGS in management [72, 76, 81]. One cost analysis forecasted that costs of WGS would gradually decrease over 10-years to a projected $1000 by September 2021 [83]. Other studies have demonstrated a wide range of costs of NGS across platforms (Table 2).

Despite the progress and promise shown in improving the cost-effectiveness of genomic sequencing in cancer care, the mixed results presented in the literature likely reflect that data from current studies assessing the costs and cost-effectiveness of NGS-based strategies are still early and relatively limited. Several systematic reviews have emphasized that there is a lack of robust published data to make an informed analysis on the cost-effectiveness of NGS and current evidence is quite heterogeneous and difficult to compare given the unclear and poor study methodologies and uncertain reproducibility of published results [71, 72]. For example, many cost estimates are based on published price lists of NGS technologies from manufacturers that often neglect the multidisciplinary nature of the work including necessary personnel, bioinformatics, and laboratory oversight [217]. Cost estimates are also limited in their applicability given that assumptions factored into cost calculations are not always transparent. Furthermore, some research institutions and manufacturers provide genomic sequencing services for profit and therefore using published pricing estimates to inform decision making in publicly-funded healthcare systems may not be directly translatable and are often not recommended.

The seemingly disparate range of costs for NGS across studies underscores the tendency to focus on expenses related to procurement and running of NGS platforms with failure to account for the real costs of the entire genome sequencing workflow, including data management and analysis [71]. The contribution of subsequent analyses to overall costs of NGS is becoming increasingly important to consider as recent projection analyses have shown that as sequencing costs continue to decrease over time, costs associated with analysis of data downstream of sequencing are expected to grow by approximately 50% between 2010 and 2020 [70, 218]. To improve assessments of the cost-effectiveness of NGS strategies, several factors need to be considered: 1.) conducting more comprehensive cost calculations with transparency of genomic sequencing that include costs of products and consumables, inpatient vs. outpatient expenses, costs based on diagnostic context (e.g., NGS to inform cancer care vs. NGS to diagnose a rare disease in genetic counseling), approach and technology used (e.g., cost per Mb and sequencing time), personnel and labor costs, costs of bioinformatics, and additional cost factors such as overhead costs, 2.) redefining the conventional $50,000 per QALY threshold to reflect higher and more contemporary cost-per-QALY thresholds, and 3.) placing value on sequencing results that could affect family members and/or economic impact of secondary findings or incidental findings [71, 82, 219, 220].

A comprehensive understanding of costs as just described is critical to our ability to assess the cost-effectiveness of NGS in cancer care; however, development of strategies to improve the costs and efficiency of precision oncology requires a greater understanding of health economics and policy, which is beyond the scope of this review. In general, as increasing commercialization and application of NGS in clinical and research settings are expected, costs for equipment and consumables may lower as a result of competition and economies of scale [84]. In the U.S., the Orphan Drug Act of 1983 (ODA) pathway, the extent that precision drugs are more likely to be biologic and require technology-intensive manufacturing, development of biomarkers and diagnostics, and costs needed to justify expected research and development expenditures are all factors critical in shaping the pricing of precision medicine [221]. Implementing financial instruments similar to mortgages that spread the costs of high-value, high-price treatment approaches over time, spreading costs over larger insurance pools or publicly financed “high-risk pools,” and creating price competition through expediting biosimilar approval, encouraging physician use of biosimilars, and stimulating brand-brand and biologic-biosimilar competition represent several proposed and potential means of offering financial relief for patients and payers of precision oncology strategies [221]. Last but not least, cancer treatment pathways represent a growing and ever-important concept with potential to incorporate molecular profiling in promoting high-value care through helping oncologists identify evidence-based treatments of greatest clinical benefit while reducing costs [23, 39, 40].

The evidence supporting the clinical benefit (effectiveness) of NGS in cancer care appears relatively more robust than those supporting its cost-effectiveness. Guidelines currently recommend molecular profiling as standard practice in the management of a growing number of cancers (Supplementary Table 1). Aside from the value to routine cancer care, NGS provides research value through expediting the detection of novel biomarkers with the potential to further improve patient outcomes in the future. Furthermore, meta-analyses of >1,000 prospective clinical trials enrolling >80,000 patients with solid tumors and hematologic malignancies have shown superior outcomes in those with personalized treatment strategies over those in nonpersonalized management arms (Table 3). Mature and final results from a number of cancer clinical trials matching targeted therapy to genomic profiling are eagerly awaited to see if further support for precision oncology is provided [9–17]. Again, clinical pathways in cancer represent a potential but important avenue to maximize clinical benefit through incorporation of genomic sequencing [39, 40].

To further enhance our ability to achieve clinical benefit or effectiveness in cancer care through NGS, we should understand that not all NGS platforms are equivalent in performance and applicability in the clinic. Currently there are several types of NGS platforms including single-gene tests, targeted gene panels utilizing polymerase chain reaction (PCR)-based techniques or DNA captures, WES, and WGS [77, 84]. Differences in coverage across platforms have been the subject of early studies [84, 222, 223]. NGS targeted panels have been reported to have approximately 4- to 5-fold greater coverage than WES, while another report illustrates the average coverage for targeted panels to be 100X, 70X for WES, and 30X for WGS [84, 223]. For high-quality genome data, many commercial entities recommend 30X coverage for WGS to represent a contemporary benchmark [82, 84]. Despite typically greater coverage for targeted panels, variability in the coverage of designed probes targeting a genomic region of interest have been shown across commercially available target enrichment methods [48]. Additionally, missed opportunities for treatment have been shown in cross-comparisons of NGS panels where instances of missed germline mutations and copy number variation detection occurred [49].

Recent results suggest that for Mendelian diseases and certain cancers WES may achieve a diagnostic yield similar to panel-based targeted sequencing though a higher false negative rate should be considered for WES use in cancer [224, 225]. In addition, one investigation has demonstrated a high concordance with WES across institutions [226]. On the contrary, comparison of WES to WGS may result in small differences in diagnostic yield given that WES accounts for all protein-coding regions in which 85% of all mutations are believed to occur [84]. Evidence suggests that single-gene tests are best served in clinical scenarios with minimal locus heterogeneity while NGS panels are less useful in those with extreme heterogeneity where targeted panels identify fewer actionable alterations than other platforms – in instances of rare diseases or in patients with an abnormal or unknown phenotype, WGS may provide the diagnostic solution [82, 223]. Certainly, WGS has established a diagnostic role in medical genetics, typically after first-tier testing [223]. However, WGS has been associated with a greater potential to detect IFs compared to targeted panels (less likely) and single-gene tests (no IFs), which given the increased costs and possible complications incurred from further diagnostic work-up prohibits its widespread and first-tier application except in certain conditions [82, 223]. One group has recently proposed a role for WGS as a first-tier genetic test given the improved diagnostic yield compared with targeted gene sequencing panels and WES in a pediatric population [227]. For cancer care, WGS may ultimately generate more cost savings if used as a first-tier strategy given the potential for earlier diagnoses and avoidance of ineffective therapy as well as identification of resistance mechanisms to often expensive therapeutic agents [83].

Along these latter lines, analysis of cell-free circulating tumor DNA (ctDNA) represents a promising development that can optimize clinical benefit in cancer treatment given its ability to detect resistance mutations and serially monitor a tumor’s molecular profile to various pressures including systemic therapy [228–230]. Extension of targeted deep sequencing that allows for detection of DNA rearrangements and copy number variations to whole-exome or whole-genome approaches in plasma has afforded predictive and prognostic information in cancer patients. In addition, ctDNA analysis is relatively simple and noninvasive compared to tissue biopsy and the ability to collect serial assays and provide comprehensive molecular profiling over time can address concerns regarding genetic heterogeneity in tumor specimens procured from tissue biopsies.

In the current landscape, targeted gene panels and single-gene testing are recommended by national guidelines (NCCN) and are more routinely used in the diagnostic evaluation and management for several cancers while WES and WGS are not yet as incorporated into routine clinical practice in cancer care [36–38]. With the continued development and decrease in costs of NGS technologies, the streamlining of WGS into a single laboratory workflow and one-time test providing the basis for lifelong follow-up that replaces other sequencing tests is within reach in the foreseeable future [223]. Notably, as NGS becomes more entrenched in routine cancer care, further development of NGS targeted panels should undergo rigorous validation that requires adequate training of pathologists and refinement of international laboratory standards [231]. Similar stringent guidelines for validation of WES and WGS will be required as well, should these platforms become further implemented in cancer care and research [232].

Targeted gene panels and WES are relatively less costly than WGS, although this price gap is narrowing (Table 2). Targeted gene panels often require complementary assays to detect duplications and deletions and Sanger sequencing for confirmation while WES often requires Sanger sequencing for confirmation as well [223]. Furthermore, a major obstacle to the routine clinical application of NGS has been interpretation of the large number of sequencing variants and variants of unknown significance (VUS) of which published results to help assess variant pathogenicity provided by public and commercial databases can often contain ambiguous or insufficient information that may potentially lead to overassessment of pathogenicity and misdiagnosis [223]. Of the number of VUS that are potentially detected, single-gene tests detect the fewest followed by targeted gene panels and WES which can detect large numbers of VUS. Ultimately, the decision to proceed with a clinical sequencing platform and method is dependent on numerous considerations including, but not limited to, required turnaround time, samples to be tested, type and complexity of the genetic variants to be assessed, required sensitivity, degree of bioinformatics support, infrastructure, and resources available in the laboratory (particularly computational resources), expected volume of testing, and overall costs per sample [84, 233].

Although molecular profiling can improve safety and tolerability regarding to toxicities associated with cancer therapies, financial toxicity remains a major looming challenge to achieving value-based genomics. As highlighted by a timely study, U.S. payers in general believe that NGS does not fit the definition of medically necessary and is considered experimental or investigational, NGS represents a misalignment to the single test/single result contemporary coverage approach, current evidence methods proposed for NGS do not fit payers’ evidentiary standards, and that issues regarding the adoption and implementation of NGS in cancer care will arise given the potential for departure from standard care protocols, lack of transparency on NGS application, and lack of competent infrastructure [216].

The authors in this study propose 2 general approaches to increase payer coverage: redefine NGS to satisfy the current coverage and evidence framework or redefine the current coverage and evidence framework to satisfy NGS. For within the current coverage framework approach, evidentiary challenges, for example, could be addressed by collaboration with the health technology and pharmaceutical industry, physicians and healthcare providers, policy-makers, and stakeholders to agree on novel research methods and develop corresponding evidence [216]. Spreading the costs of high-value, high-price treatment approaches over time, spreading costs over larger insurance pools or publicly financed high-risk pools, and creating price competition in precision oncology are also potential strategies to provide financial relief for patients and payers within the current framework [221]. Furthermore, success of basket trials or trials investigating targeted therapy matched by genomic profile can reduce both the cost and length of trials allowing more drugs to become more commercially viable that can lead to more innovation and competition [221]. Payers have shown enthusiasm regarding the value of pathways as they reduce unwarranted variation in care and improve adherence to evidence-based medicine; further development of clinical pathways that incorporate genomics-based treatment represents an additional strategy within the current framework to improve payer coverage [39, 40]. Pan-cancer NGS applications are becoming more common and provide rationale for off-label drug use allowing for payment that can be continued on exception bases as another method to reduce financial toxicity [216].

The second approach of redefining the current coverage and evidence framework to satisfy NGS is undoubtedly more complex [216]. Here, the authors comment that this approach would require collaboration with stakeholders to explicitly identify and define coverage disruptive features of NGS, modify the evidentiary framework including evidence research methods and approach to assessing evidence, and adjust the current coverage framework to align with the evidentiary framework and permit incorporation of NGS benefits. In essence, proactive multidisciplinary efforts to define methods of evidence generation, the direction for which NGS development should proceed, and implementation into coverage policy are fundamental aspects to this approach that although seemingly uncertain can provide unprecedented benefits and reduce financial toxicity in the era of precision oncology.

Lastly, our analysis is based primarily on available data, especially from the U.S. It will now be important to accumulate and analyze data to assess the value of genomics in cancer care from other countries. We hope that in the future there will ultimately be international guidelines for genomics in oncology.

## SUPPLEMENTARY MATERIALS TABLE




