# Supplementary material for: Value-based genomics
Source: Oncotarget. 2018 Jan 30;9(21):15792–815. doi: 10.18632/oncotarget.24353 (PMC5884665; doi:10.18632/oncotarget.24353)
Supplement: Supplementary file 1 [file oncotarget-09-15792-s001.pdf]

Value-based genomics

SUPPLEMENTARY MATERIALS

Supplementary Table 1: National Comprehensive Cancer Network (NCCN) recommended biomarker strategies in select solid tumors and potential biomarkers undergoing investigation using next-generation sequencing. See Supplementary_Table_1
