# Supplementary material for: Value-based genomics
Source: Oncotarget. 2018 Jan 30;9(21):15792–815. doi: 10.18632/oncotarget.24353 (PMC5884665; doi:10.18632/oncotarget.24353)
Supplement: Supplementary file 2 [file oncotarget-09-15792-s002.docx]

| **Supplementary Table 1:** National Comprehensive Cancer Network (NCCN) recommended biomarker strategies in select solid tumors and potential biomarkers undergoing investigation using next-generation sequencing | | | |
| --- | --- | --- | --- |
| Solid tumor | NCCN recommendations | Investigational biomarkers | Ref. |
| Breast | ER/PR status (IHC) to guide hormonal therapy  HER2 status (IHC/FISH) to guide HER2-directed therapy  BRCA 1/2 status to guide olaparib therapy, risk reducing surgery, genetic counseling | Activating HER2 mutations (not detectable by IHC/FISH), BRAF, ESR1: predictive  CNVs in CCND1-3, CDK4/6, FGFR1-4, AKT1-2/PIK3CA/PTEN pathways, TP53, MLL3, NOTCH2: prognostic  HLF, CXCL13, SULT1E1, GBP1, FGFR: prognostic  BRCA 1/2: predictive  CD8, PD-1, PD-L1, MKI67, CDC20, NUF2, KIF2C, CENPF, EMP3, TYMS, ANGPTL4, FGFR4, VEGFA, ESPL1, SPAG5, MKI67, PLK1, PGR: predictive  TP53: predictive | [102-110] |
| Colorectal | Extended RAS status (KRAS and NRAS) to guide anti-EGFR therapy  BRAF status to guide anti-EGFR therapy  MSI/MMR status to guide PD-1 inhibitor therapy | HER2 overexpression or amplification: predictive  HER2 and HER3 mutations: predictive  POLE, PD-1, PD-L1, CTLA-4: prognostic and predictive  PIK3, PTEN, MEK/ERK, AKT, RNF43, SMAD, APC, TP53, FBWX7, CCL5, CCR5, ARAF, MET: prognostic and/or predictive  Tumor mutational burden: predictive  LEFTY, Nodal, ACVR2B: prognostic | [111-115] |
| Gastroesophageal | HER2 overexpression (IHC or FISH) to guide HER2-directed therapy  MSI/MMR status to guide PD-1 inhibitor therapy | HER2 extracellular domain mutations: predictive  KRAS and EGFR amplifications: predictive  ERCC1: predictive  PD-1, PD-L1, POLD1, POLE, tumor mutational burden: prognostic and predictive  RICTOR, TSC 1/2, TP53, MET amplification or overexpression, PIK3CA, MEK/ERK: predictive  FGFR2 amplification: predictive | [116-122] |
| Hepatobiliary | None | APC, ATM, BRCA 1/2, CDKN2A, BARD1, BLM, CHEK2, FAM175A, FH, MITF, MUTYH, NBN, NF1, PALB2, PMS2, POLE, RAD50, TSC: predictive and prognostic  c-MET, MAPK, MTOR, or FGFR: predictive or prognostic  Tumor mutational burden, DNA repair pathway (MSH6, BRCA, ATM, MLH1, MSH2), IDH TP53, PIK3, MTOR, MET: prognostic and/or predictive  BRAF: predictive | [123-127] |
| Pancreatic | MSI/MMR status to guide PD-1 inhibitor therapy | APC, ATM, BRCA 1/2, CDKN2A, BARD1, BLM, CHEK2, FAM175A, FH, MITF, MUTYH, NBN, NF1, PALB2, PMS2, POLE, RAD50, TSC: predictive or prognostic  DNA repair pathway (MSH6, BRCA, ATM, MLH1, MSH2): predictive  KRAS, TP53, CDKN2A, SMAD4: prognostic or predictive  IDH, ERBB2: predictive | [128-130] |
| Gynecologic | Germline BRCA 1/2 status to guide risk reducing surgery, genetic counseling, PARP inhibitor therapy  Somatic BRCA 1/2 status to guide PARP inhibitor therapy  MSI/MMR status to guide genetic counseling, PD-1 inhibitor therapy | PD-L1, POLE, POLD1: predictive  KRAS, MAPK1, PIK3CA ERBB2, ERBB3, OVOL1, PD-L1, TGFB1, ENO1, MYC, NF1, BRCA-wild-type LOH, CCNE1, AKT2, BRAF, PTEN, TP53, tumor mutational burden, MSI: prognostic, or predictive  DNA homologous recombination genes (ATM, BARD1, BRIP1, CHEK1, CHEK2, FANCA, MRE11, NBN, PALB2, PTEN, RAD51D, RAD51C, p53): prognostic and/or predictive BAIAP3, PLCB1, IL1R1, NOS3, RAD50, BMP7, FGF18, WNT7A, cell cycle genes, FGF18, CCND1, HIST1, HOXA11, LEFTY2 ,SFRP4: prognostic | [131-143] |
| Prostate | BRCA, MLH1, MSH2, MSH6, or PMS2 status to guide cancer screening | DNA repair pathway (ATM, BRCA 1/2, CHEK2, RAD51D, PALB2, ATR, NBN, PMS2, GEN1, MSH2, MSH6, RAD51C, MRE11A, BRIP1, FAM175A, FANCA, FANCM, POLH, BARD1, CDK12, CHEK1, FANCL, PPP2R2A, RAD51 (B,C,D), RAD54L): predictive  ARV7, HSD3B1, genomic prostate score (GPS), TP53, RB1, PTEN, WNT/B-catenin pathway, ZFP36: prognostic and/or predictive- TMPRSS2-ERG, TMPRSS2-ETV1 fusions: diagnostic or prognostic | [144-152] |
| Kidney, germ cell/testicular, bladder | None | MET, TERT, CDKN2A/B, EGFR, NF2, FH, SETD2, TFE3, SWI/SNF complexes, RAS/RAF, PI3K/mTOR: actionable  APC, ATM, BRIP1, BRCA1/2, CHEK2, FH, BAP1, MET, SDHA/SDHB, VHL, MLH1, MSH2, MSH6, NBN, BRCA2, PALB2, PBRM1, RAD51C, TP53: diagnostic and/or predictive  16-gene recurrence score, ATM, DLL3, ERBB2, HPGD, PD-L1, PRC1, NCAM1, RB1: prognostic  18-gene expression profile, PD-L1, MDM2, MSI, tumor mutational burden, TP53: predictive  Chromosome 12p:CLEC6A-CLEC4D, ETV6-RP11-434C1.1: diagnostic | [153-166] |
| Lung | EGFR, ROS1, ALK, BRAF to guide TKI therapy  PD-L1 expression to guide PD-1 inhibitor therapy  T790M status to guide osimertinib therapy | HER2, PIK3CA, AXL, FGFR, NTRK1, NTRK2, NTRK 3, RET, MET (amplification and exon 14), IDO1, RB1 PTCH1, SMO, EPHR, RON: predictive, and/or prognostic  BIM, SMAD4, DNMT3A, GNAS, ATM, KIT, PIK3CA, PTEN, mTOR, TET2; CNVs in CDK4, MDM2, MYC, RICTOR, ERBB2, CRKL, YES1: predictive  Tumor mutational burden, CNVs in STK11/LKB1, JAK2, CD274, TP53: prognostic and/or predictive  TP53, RB1, MYC/MYCL1/MYCN, FGFR1 copy number gains, PI3K/AKT/mTOR pathway mutations (PIK3CA ,PTEN, TSC2): predictive  TP53, RB1, MYC/MYCL1, MLL2, LRP1B, PTEN, STK11, KEAP1: diagnostic | [167-179] |
| Head and neck | None | Tumor mutational burden, IDO1, PD-L1: predictive  PI3K, HER2, FGFR, CDKN2A, PTEN, NOTCH1, NRAS, ZNFR, TP53, ADCY2, TGFBR2: predictive and/or prognostic | [180-187] |
| Melanoma | BRAF status to guide BRAF inhibitor therapy  KIT status to guide TKI therapy  PD-L1 expression to guide checkpoint inhibitor therapy (non-uniform consensus) | PIK3CA, FBXW7, NF2, RB1, SMAD4, FBXW7, NRAS, KRTAP4-7, KRTAP4-5, MUC2, MUC21: prognostic and/or predictive  EZH2, LAG-3: actionable  Tumor mutational burden, CNVs and alterations in PTEN, JAK2, B2M: predictive | [188-194] |
| Sarcoma | PDGFRA, KIT, SDH status ancillary diagnostic tests for GIST  CD117, DOG-1, CD34 status ancillary diagnostic tests for GIST | EWRS1, ETV1, NY-ESO-1: diagnostic or prognostic  BRAF, NF1, TP53, MAX, MLL2, SETD2, PIK3CA, TSC1, RB1, SETD2, PTEN, ANKRD11, TP53, TSC1, CDKN2A, ZNF717, SPDYE1, CRIPAK, CNVs in NEURL1B, FLG, GSTT1, CEACAM, ATRX, KMT2D, ATM, ERBB4, ARID1A, MDM2, CDK4, GLI1, MAP2KA, TERT, CDKN2B, AKT, ESR1, BRCA, NTRK, PTCH1, SMARCB1, TOP2A , TOPO1 , AR, EGFR, TUBB3, MGMT, TS, RRM1, ERCC1: actionable  Tumor mutational burden, MMR, PD-L1, FAS: predictive | [195-204] |
| Brain | MGMT, 1p19q, IDH 1/2 status (FISH, PCR) prognostic and to guide chemotherapy | ATRX, TERT, SDF1/CXCR4: prognostic and/or predictive  EGFR, PD-L1, VEGFA, RRM2, MAPK9, XIAP, and fusion genes involving NTRK, MET, EGFR, FGFR3, BRAF, PDGFRA: actionable  Tumor mutational burden: predictive | [205-212] |
| ER, estrogen receptor; PR, progesterone receptor; IHC, immunohistochemistry; HER2, human epidermal growth factor receptor 2; FISH, fluorescent in situ hybridization; CNVs, copy number variations; EGFR, epidermal growth factor receptor; MSI, microsatellite instability; MMR, mismatch repair; PD-1, programmed death 1; PARP, poly ADP-ribose polymerase; TKI, tyrosine kinase inhibitor; PD-L1, programmed death ligand 1; MGMT, O6-methylguanine-methyltransferase; IDH, isocitrate dehydrogenase; PCR, polymerase chain reaction; GIST, gastrointestinal stromal tumor; PDGFRA, platelet-derived growth factor receptor alpha; SDH, succinate dehydrogenase; DOG-1, discovered on GIST-1 | | | |
